# Sea anemones modify their hiding time based on their commensal damselfish

**DOI:** 10.1098/rsos.160169

**Published:** 2016-08-24

**Authors:** Alexandra N. Lim, Justin A. De La Guerra, Daniel T. Blumstein

**Affiliations:** Department of Ecology and Evolutionary Biology, University of California, 621 Young Drive South, Los Angeles, CA 90095-1606, USA

**Keywords:** hiding time, antipredator, defence, dynamic mutualism, anemones, anemonefish

## Abstract

Animals often retreat to refugia when alarmed and the time they spend hiding reflects an economic decision that trades off reducing predation risk with other beneficial activities. Typically, refugia such as burrows are static, but some refugia are dynamic. For species with defensive mutualisms, hiding might be contingent on their mutualist's behaviour. We disturbed and quantified hiding time in magnificent sea anemones, *Heteractis magnifica,* and their associated domino damselfish, *Dascyllus trimaculatus*. We found that sea anemone hiding behaviour was dependent on the number and behaviour of their commensal fish: anemones emerged sooner when they had more associated fish and faster returning fish. Together, these results demonstrate that hiding behaviour can be influenced by the behaviour of a commensal; such dynamic mutualisms may be found in other systems.

## 1. Introduction

Prey that do not escape predators or that suffer non-fatal injuries incur a cost, and there are a variety of complex antipredator adaptations to reduce these costs [1]. For example, many animals hide in protective refuges for variable lengths of time. Fiddler crabs, *Uctea lactea perplexa*, retreat to burrows, and polychaete worms, *Serpula vermicularis*, retreat into their calcareous tubes [2]. Variation in hiding, like many other antipredator behaviours, has an economic logic. The optimal hiding time model [3] suggests that antipredator hiding time decisions are based on a balance between predation risk and the cost of refuge use. Once hidden, prey can emerge when the predator is still present and be killed, or stay in the refuge long after the predator is absent and lose foraging opportunities [4].

A defining characteristic of previous studies has been that they involved static hiding systems where organisms sought shelter in unchanging refugia. Examples of static terrestrial refugia include trees, cliff faces, burrows, thick vegetation and rock talus [5]. However, there are some systems where shelters are dynamic rather than static. One such system is the anemone–anemonefish mutualism. In these systems, host anemones create dynamically changing shelters for anemonefish, whereas fish defend their host anemones from predation [6].

Sea anemones avoid predators by tensing or withdrawing their tentacles into the upper column of their body cavity and effectively hiding [7]. When closed, commensal anemonefish hide among rocks or below the skirt of their host anemone. Unlike other static systems, the antipredator hiding behaviour of anemones and anemonefish are highly dependent on one another and can be described as a dynamic protection mutualism. Their dynamic behaviour makes this an excellent system to ask whether variation in fish behaviour affects anemone hiding.

## 2. Material and methods

### 2.1. Study site and subjects

We studied magnificent sea anemones, *Heteractis magnifica,* attached to individually mapped coral bommies, and domino damselfish, *Dascyllus trimaculatus*, between 22 January and 30 January 2016 in Opunohu and Ha'apita Beaches, Mo'orea, French Polynesia (17.53°S, 149.83°W). All Opunohu anemones (*n* = 24) had commensal anemonefish, whereas Ha'apita anemones (*n* = 12) had none. When bommies had more than one anemone, we randomly selected one of the presumed clones for study [8]. We counted the total number of anemonefish associated with a bommie and categorized them into three size classes: small (less than 2 cm), medium (2–5 cm) and large (more than 5 cm) maximum length. Anemones fire either or both offensive or defensive nematocysts in response to biological stimulation [9]. We therefore collected an abundant macroalgae, *Turbinaria ornata,* and systematically disturbed the anemones and their associated fish by swiping the anemone with algal thalli seven times. The first researcher (A.N.L.) disturbed the anemone and retreated 1.5 m away; a distance that pilot observations showed would not interfere with fish behaviour. Anemones responded to rubbing by tensing and retracting their tentacles and occasionally lifting their skirt off the substrate. The second researcher, at a distance of 1.5 m, video recorded (Fujifilm FinePix XP80 or Olympus Tough digital cameras) all experiments 30 s prior to disturbance until the anemone was fully relaxed and all anemonefish had returned. We measured, from the video, the latency to initial anemone movement as the time from disturbance until first movement of tentacles, and the latency to total anemone relaxation as the time from the disturbance until the subject was freely moving all tentacles and its skirt was completely relaxed. We also measured latency to first fish return and latency to all fish return as the time from when all fish hid until the first fish and then all fish returned to the anemone. All anemones were 1–2 m deep. To obtain a better estimate of the repeatability of hiding time and fish counts, we resampled 23 marked Opunohu individuals three times—once every other day.

### 2.2. Statistical analyses

Using the full dataset of the day one experimental manipulations on each anemone, we fitted general linear models (GLMs) to explain variation in the latency to initial anemone movement following disturbance as well as the latency to full anemone relaxation as a function of total number of fish. Using the Opunohu dataset of day one experimental manipulations, we fitted linear models to explain anemone behaviour as a function of total number of fish, latency to first anemonefish return and latency to all anemonefish return.

To quantify the consistency of our estimates of both fish counts and of hiding time, we fitted a series of linear mixed-effects models on the Opunohu data. We first fitted a random intercept model where we explained variation in the number of fish or hiding time as a function only of anemone ID. This permitted us to calculate the intraclass correlation coefficient, a measure of repeatability for the number of fish, latency to anemone initial movement and latency to total relaxation. We then fitted a random intercept model and a random slope model with a set of fixed effects. In all cases, we included trial number, total number of fish, latency to first fish return and latency to all fish return as fixed effects. We compared the mixed models to a GLM with no random effects using likelihood ratio tests (LRTs).

We log_10_-transformed total number of fish, latency to initial anemone movement, latency to total anemone relaxation, latency to first fish return and latency to all fish return. Analyses were conducted in R v. 3.2.3 [10]. We used Deducer [11] to fit the GLMs, the R packages lme4 [12] for the mixed-effects models and lmeTest [13] to calculate *p*-values for the mixed models. For the GLMs, we report type III sums of squares and adjusted *R*^2^-values. We calculated likelihood ratio tests using lme4 and RSLsim [14]. Our residuals were approximately normally distributed. We plotted residuals versus fitted values and found two to three outliers. While there was no reason to remove them, we did so, and recalculated the models. The overall model fit improved after removing outliers, but there was no change in the general conclusions from the models with the outliers. We thus report the results of the dataset including statistical outliers.

## 3. Results

### 3.1. Latency to first movement following disturbance

**Figure 1.**
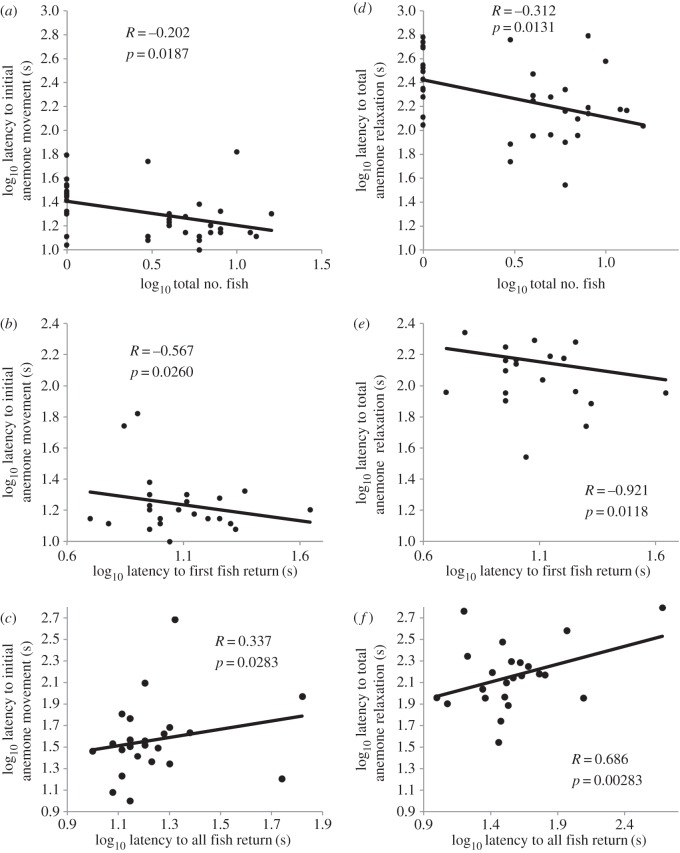
Log_10_ latency for initial movement following disturbance as a function of (*a*) log_10_ total number of fish, (*b*) log_10_ time for first anemonefish to return and (*c*) log_10_ time for all fish return. Log_10_ latency to total anemone relaxation following disturbance as a function of (*d*) log_10_ total number of fish, (*e*) log_10_ time for first anemonefish to return and (*f*) log_10_ time for all fish return.

Anemones varied in their number of commensal fish. While anemones at Opunohu had at least some commensal fish, anemones at Ha'apita had none. Using the full dataset, we found that anemones with more commensal anemonefish first moved sooner after experimental disturbance (estimate = −0.202, *p* = 0.0187; figure 1*a*). This model explained 12.7% of variation in anemone hiding time.

Focusing only on the subset of anemones with commensal fish at Opunohu, and after explaining variation accounted for by total number of fish (estimate = −0.206, *p* = 0.319), latency to first fish return (estimate = −0.567, *p* = 0.0260) and latency to all fish return (estimate = 0.337, *p* = 0.0283), we found that anemones with a shorter latency to first fish return had a longer latency to initial tentacle movement (figure 1*b*). Anemones with fish that all returned sooner, first moved their tentacles sooner (figure 1*c*). This model explained 15.4% of variation in anemone hiding time (*p* = 0.107).

For Oponohu data, we explored how fish size influenced hiding time by modelling latency to first move as a function of either the number of small (estimate = −0.158, *p* = 0.158), medium (estimate = −0.225, *p* = 0.0706) or large (estimate = −0.578, *p* = 0.002) fish. Thus, the number of large fish best explained variation in anemone hiding behaviour.

### 3.2. Latency to full relaxation following disturbance

In the full dataset, we found that anemones with more commensal anemonefish fully relaxed sooner (estimate = −0.312, *p* = 0.0131; figure 1*d*). The model explained 14.33% of variation in anemone hiding time.

Focusing only on the subset of anemones with commensal fish at Opunohu, and after explaining variation accounted for by total number of fish (estimate = −0.257, *p* = 0.375), latency to first fish return (estimate = −0.921, *p* = 0.0118), and latency to all fish return (estimate = 0.686, *p* = 0.00283), we found that anemones with shorter latency to first fish return had longer latency to relax (figure 1*e*). Anemones with shorter latency to all fish return fully relaxed sooner (figure 1*f*). This model explained 30.4% of variation in anemone hiding time (*p* = 0.0194).

For Oponohu data, we explored how fish size influenced hiding time by modelling latency to total relaxation as a function of either the number of small (estimate = −0.276, *p* = 0.0918), medium (estimate = −0.321, *p* = 0.0805) or large (estimate = −0.748, *p* = 0.0066) fish. The number of large fish best explained variation in anemone latency to total relaxation.

### 3.3. Consistency in anemone response behaviour

**Figure 2.**
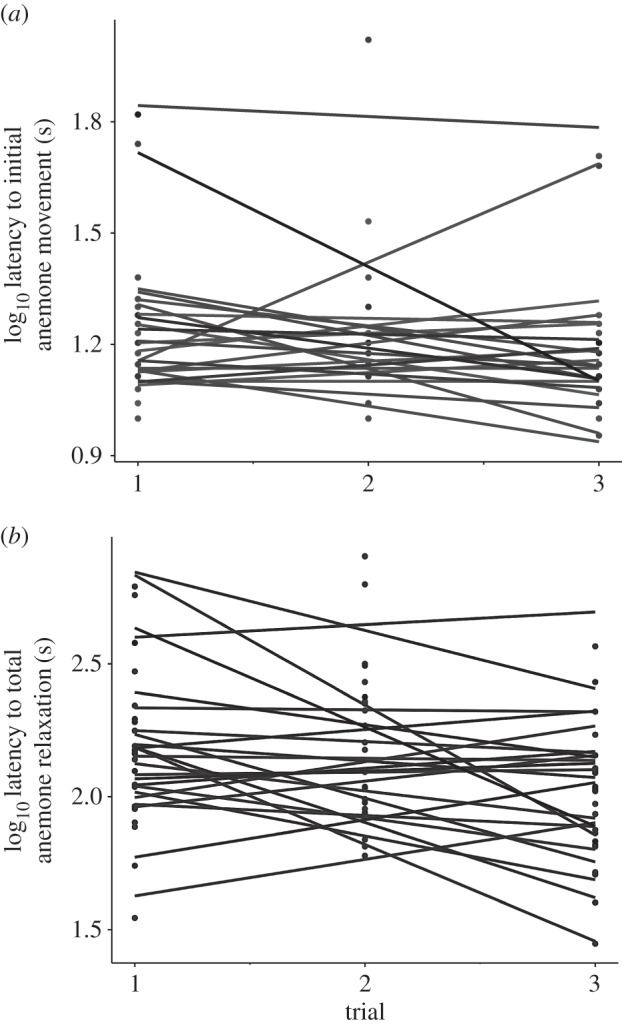
Repeatability of (*a*) anemone latency to first movement and (*b*) latency to total relaxation over three separate experimental disturbances. A random intercept model best explained variation in hiding time.

Fish counts were highly repeatable over 3 days (ICC = 0.84). After accounting for variation explained by our fixed factors, individual anemones had significantly different latencies to first movement (*p* = 0.0291; figure 2*a* and electronic supplementary material, table S1*a*) but they did not differ in their slopes (*p* = 0.0556; electronic supplementary material, table S1*b*). This random intercept model on latency to first movement was superior to a GLM with no random effects (LRT = 17.6, *p* = 2.2 × 10^−16^; electronic supplementary material, table S2*a*). Similarly, after accounting for variation explained by our fixed factors, individual anemones had significantly different latencies to total relaxation (*p* = 0.000488; figure 2*b* and electronic supplementary material, table S1*a*) but they did not differ in their slopes (*p* = 6.97 × 10^−15^; electronic supplementary material, table S1*b*). This random intercept model on latency to total relaxation was superior to a GLM with no random effects (LRT = 7.10, *p* = 0.0023; electronic supplementary material, table S2*b*).

## 4. Discussion

We found that variation in anemone behaviour was contingent on number and behaviour of anemonefish on two timescales: both the short term and the long term. Anemones moved and fully relaxed sooner when associated with fish with shorter latencies to return. Likewise, anemones associated with more commensal fish moved and reached full relaxation sooner following disturbance. Thus, variation in anemonefish behaviour explains variation in anemone hiding time.

Strictly, because all anemones at the Opunohu site were associated with fish, while none at the Ha'apiti site were, it was not possible to statistically isolate any effects of site on anemone response. We do not know why there were no fish associated with the anemones at the Ha'apiti site. Both the anemones, and the site itself appeared healthy. Future studies, at more sites, will be required to better understand the effects of site variation on anemone antipredator behaviour.

Anemones vary in their size, and this could influence the latency it takes them to relax. A set of complementary analyses (electronic supplementary material, table S3) revealed that for almost all of the analyses, the addition of maximum anemone diameter (a measure of size) as a covariate either failed to improve the model, had no significant effect on latency to relax, or eliminated the significance of the models entirely. From this, we conclude that anemone size has a limited effect on the time it takes them to relax following disturbance.

These results demonstrate for the first time that variation in hiding behaviour can be influenced by the variable behaviour of a commensal species. While there are a number of well-studied protective mutualisms including ants and their plants, and squid and their bioluminescent bacteria, no prior studies have analysed the dynamics of hiding in the protected species [15]. Interestingly, it seems that the number of large fish may drive this relationship, because our analysis suggests that only the number of large fish explained variation in hiding time. A previous study found that anemone growth rate depended on the size and number of anemonefish and that anemones with more large fish had higher growth rates [16]. This dynamic protective mutualism appears more complex, because the benefits and costs may vary as a function of fish size to both the anemone and the fish themselves.

Dynamic mutualisms may exist in other systems. Future studies should evaluate various marine and terrestrial mutualisms to better understand how defensive behaviour can dynamically change depending on the behaviour of a mutualistic partner.

## Supplementary Material

Supp Material.

## Data accessibility

The dataset supporting this article is provided as electronic supplementary material.
