# Supplementary material for: Sea anemones modify their hiding time based on their commensal damselfish
Source: R Soc Open Sci. 2016 Aug 24;3(8):160169. doi: 10.1098/rsos.160169 (PMC5108943; doi:10.1098/rsos.160169)
Supplement: Supp Material. [file rsos160169supp1.docx]

**Supplementary tables**

**Table S1.** (a) Fixed effects from the random intercept model explaining latency to first anemone movement and latency to total anemone relaxation. (b) Fixed effects from the random slope model explaining latency to first anemone movement and latency to total anemone relaxation. Total number of fish, latency to first fish return, and latency to all fish return have been log_10_ transformed. Likelihood ratio tests show that the random intercept models are better than random slope or null models with ID and the random effects.

| (*a*) | | | |
| --- | --- | --- | --- |
| **random intercept model: latency to first anemone movement** | | | |
| fixed effects | estimate | standard error | p-value |
| total number of fish | -0.132 | 0.137 | 0.339 |
| latency to first fish return | 0.0126 | 0.0926 | 0.893 |
| latency to all fish return | 0.156 | 0.070 | 0.0297 |
| trial | -0.0209 | 0.0195 | 0.290 |
| **random intercept model: latency to total anemone relaxation** | | | |
| fixed effects | estimate | standard error | p-value |
| total number of fish | -0.394 | 0.189 | 0.0430 |
| latency to first fish return | -0.238 | 0.141 | 0.0975 |
| latency to all fish return | 0.398 | 0.107 | 0.000392 |
| trial | -0.0711 | 0.0315 | 0.0283 |
| (*b*) |  |  |  |
| **random slope model: latency to first anemone movement** | | | |
| fixed effects | estimate | standard error | p-value |
| total number of fish | -0.191 | 0.136 | 0.166 |
| latency to first fish return | -0.0221 | 0.0907 | 0.809 |
| latency to all fish return | 0.172 | 0.0685 | 0.0146 |
| trial | -0.0235 | 0.0219 | 0.293 |
| **random slope model: latency to total anemone relaxation** | | | |
| fixed effects | estimate | standard error | p-value |
| total number of fish | -0.413 | 0.188 | 0.0334 |
| latency to first fish return | -0.245 | 0.139 | 0.0820 |
| latency to all fish return | 0.380 | 0.105 | 0.000573 |
| trial | -0.0738 | 0.0346 | 0.0424 |

**Table S2.** Linear mixed effects models fitted in R with random and fixed effects.

| Description | Model | AIC |
| --- | --- | --- |
| (*a*)  General Linear Model (fixed effects only) | log_10_ latency to first movement ~ log_10_ fish count log_10_ latency first fish return + log_10_ latency all fish return | -29.055 |
| Random Intercept Model | log_10_ latency to first movement ~ (1 \| anemone) | -41.867 |
| Random Intercept Model (with fixed effects) | log_10_ latency to first movement ~ log_10_ fish count + log_10_ latency first fish return + log_10_ latency all fish return + trial + (1 \| anemone) | -44.652  *,† |
| Random Effects Model | log_10_ latency to first movement ~ log_10_ fish count + log_10_ latency first fish return + log_10_ latency all fish return + trial + (1 + trial \| anemone) | -42.165 |
| (*b*) | | |
| General Linear Model (fixed effects only) | log_10_ latency full ~ log_10_ fish count + log_10_ latency first fish return + log_10_ latency all fish return | 17.476 |
| Random Intercept Model | log_10_ latency full ~ (1 \| anemone) | 24.429 |
| Random Intercept Model (with fixed effects) | log_10_ latency full ~ log_10_fish count + log_10_ latency first fish return + log_10_ latency all fish return + trial + (1 \| anemone) | 12.378  **, †† |
| Random Effects Model | log_10_ latency full ~ log_10_ fish count + log_10_ latency first fish return + log_10_ latency all fish return + trial + (1 + trial \| anemone) | 15.228 |

* = comparison to basic random intercept p = 0.0291

† = comparison to basic linear model p < 2.2e-16

** = comparison to basic random intercept p = 0.0005

†† = comparison to basic linear model p = 0.0023

**Table S3.** The effects of anemone size (estimated as maximum diameter) on anemone responses. a) effect of addition of anemone size on responses studied with general linear models; b) effect of the addition of anemone size on the linear mixed effects models.

a) General Linear Models

| Model | Estimate | p-value |
| --- | --- | --- |
| log_10_ latency to first movement ~ log_10_ fish count  log_10_ anemone size | - 0.193  0.152 | 0.266  0.468 |
|  |  |  |
| log_10_ latency to first movement ~ log_10_ latency first fish return  log_10_ anemone size | - 0.223  - 0.105 | 0.288  0.745 |
|  |  |  |
| log_10_ latency to first movement ~ log_10_ latency all fish return | 0.116 | 0.349 |
| log_10_ anemone size | 0.256 | 0.572 |
| log_10_ latency full ~ log_10_ fish count  log_10_ anemone size | - 0.277  0.616 | 0.0214  0.0381 |
| log_10_ latency full ~ log_10_ latency first fish return  log_10_ anemone size | - 0.164  0.288 | 0.613  0.572 |
| log_10_ latency full ~ log_10_ latency all fish return  log_10_ anemone size | 0.355  0.474 | 0.0515  0.299 |

b) Mixed Effects Models

| Description | Model | AIC |
| --- | --- | --- |
| (*i*)  Random Intercept Model | log_10_ latency to first movement ~ log_10_ anemone size + (1 \| anemone) | -40.233 |
| Random Intercept Model (with fixed effects) | log_10_ latency to first movement ~ log_10_ anemone size + log_10_ fish count + log_10_ latency first fish return + log_10_ latency all fish return + trial + (1 \| anemone) | -42.660 |
| Random Effects Model | log_10_ latency to first movement ~ log_10_ anemone size + log_10_ fish count + log_10_ latency first fish return + log_10_ latency all fish return + trial + (1 + trial \| anemone) | -40.209 |
| (*ii*) | | |
| Random Intercept Model | log_10_ latency full ~ log_10_ anemone size + (1 \| anemone) | 25.761 |
| Random Intercept Model (with fixed effects) | log_10_ latency full ~ log_10_ anemone size + log_10_ fish count + log_10_ latency first fish return + log_10_ latency all fish return + trial + (1 \| anemone) | 14.376 |
| Random Effects Model | log_10_ latency full ~ log_10_ anemone size + log_10_ fish count + log_10_ latency first fish return + log_10_ latency all fish return + trial + (1 + trial \| anemone) | 17.212 |

**Table S4.**  Data, including fish counts, latencies to initial anemone movement, latencies to total anemone relaxation, latencies to first fish return, and latencies to all fish return.

| location | trial | anem-one ID | anemone size: maximum diameter (cm) | # small fish | # medium fish | # large fish | # total fish | latency to intitial anemone movement (s) | latency to total anemone relaxation (s) | latency to first fish return (s) | latency to all fish return (s) |
| --- | --- | --- | --- | --- | --- | --- | --- | --- | --- | --- | --- |
| Opunohu | 1 | 1 | 13 | 0 | 0 | 0 | 0 | 11 | 111 |  |  |
| Opunohu | 1 | 2 | 12 | 1 | 0 | 1 | 2 | 12 | 77 | 21 | 34 |
| Opunohu | 1 | 3 | 15 | 2 | 1 | 0 | 3 | 16 | 90 | 44 | 124 |
| Opunohu | 1 | 4 | 23 | 3 | 0 | 0 | 3 | 20 | 177 | 9 | 48 |
| Opunohu | 1 | 5 | 16 | 0 | 2 | 1 | 3 | 17 | 90 | 9 | 23 |
| Opunohu | 1 | 6 | 36 | 1 | 4 | 2 | 7 | 14 | 138 | 10 | 37 |
| Opunohu | 1 | 7 | 17 | 3 | 0 | 2 | 5 | 10 | 35 | 11 | 29 |
| Opunohu | 1 | 8 | 22 | 2 | 2 | 1 | 5 | 12 | 80 | 9 | 12 |
| Opunohu | 1 | 9 | 18 | 4 | 2 | 1 | 7 | 15 | 155 | 14 | 26 |
| Opunohu | 1 | 10 | 26 | 3 | 2 | 1 | 6 | 14 | 91 | 5 | 10 |
| Opunohu | 1 | 11 | 18 | 2 | 1 | 0 | 3 | 18 | 296 | 13 | 31 |
| Opunohu | 1 | 12 | 37 | 6 | 2 | 3 | 11 | 14 | 150 | 16 | 58 |
| Opunohu | 1 | 13 | 34 | 9 | 6 | 0 | 15 | 20 | 109 | 13 | 22 |
| Opunohu | 1 | 14 | 20 | 6 | 1 | 0 | 7 | 21 | 617 | 23 | 479 |
| Opunohu | 1 | 15 | 21 | 2 | 2 | 1 | 5 | 13 | 220 | 6 | 17 |
| Opunohu | 1 | 16 | 19 | 4 | 1 | 0 | 5 | 24 | 145 | 9 | 43 |
| Opunohu | 1 | 17 | 25 | 3 | 1 | 0 | 4 | 14 | 92 | 18 | 32 |
| Opunohu | 1 | 18 | 27 | 2 | 0 | 0 | 2 | 55 | 573 | 7 | 16 |
| Opunohu | 1 | 19 | 32 | 3 | 8 | 1 | 12 | 13 | 147 | 10 | 64 |
| Opunohu | 1 | 20 | 35 | 3 | 3 | 0 | 6 | 16 | 125 | 9 | 33 |
| Opunohu | 1 | 21 | 19 | 1 | 1 | 0 | 2 | 13 | 55 | 20 | 30 |
| Opunohu | 1 | 22 | 25 | 0 | 3 | 0 | 3 | 16 | 196 | 12 | 36 |
| Opunohu | 1 | 23 | 32 | 1 | 1 | 2 | 4 | 19 | 191 | 18 | 42 |
| Opunohu | 1 | 24 | 17 | 5 | 4 | 0 | 9 | 66 | 379 | 8 | 93 |
| Ha'apiti | 1 | 25 | 34 | 0 | 0 | 0 | 0 | 13 | 600 |  |  |
| Ha'apiti | 1 | 26 | 28 | 0 | 0 | 0 | 0 | 30 | 490 |  |  |
| Ha'apiti | 1 | 27 | 23 | 0 | 0 | 0 | 0 | 31 | 268 |  |  |
| Ha'apiti | 1 | 28 | 44 | 0 | 0 | 0 | 0 | 35 | 546 |  |  |
| Ha'apiti | 1 | 29 | 36 | 0 | 0 | 0 | 0 | 29 | 311 |  |  |
| Ha'apiti | 1 | 30 | 21 | 0 | 0 | 0 | 0 | 39 | 333 |  |  |
| Ha'apiti | 1 | 31 | 39 | 0 | 0 | 0 | 0 | 28 | 129 |  |  |
| Ha'apiti | 1 | 32 | 37 | 0 | 0 | 0 | 0 | 21 | 219 |  |  |
| Ha'apiti | 1 | 33 | 61 | 0 | 0 | 0 | 0 | 20 | 351 |  |  |
| Ha'apiti | 1 | 34 | 22 | 0 | 0 | 0 | 0 | 26 | 225 |  |  |
| Ha'apiti | 1 | 35 | 35 | 0 | 0 | 0 | 0 | 62 | 505 |  |  |
| Ha'apiti | 1 | 36 | 14 | 0 | 0 | 0 | 0 | 34 | 190 |  |  |
| Opunohu | 2 | 1 | 13 | 0 | 1 | 0 | 1 | 34 | 228 | 257 | 257 |
| Opunohu | 2 | 2 | 12 | 1 | 0 | 1 | 2 | 14 | 225 | 12 | 14 |
| Opunohu | 2 | 3 | 15 | 2 | 4 | 1 | 7 | 17 | 224 | 12 | 32 |
| Opunohu | 2 | 4 | 23 | 4 | 1 | 0 | 5 | 17 | 316 | 11 | 43 |
| Opunohu | 2 | 5 | 16 | 2 | 2 | 0 | 4 | 16 | 124 | 6 | 13 |
| Opunohu | 2 | 6 | 36 | 2 | 2 | 1 | 5 | 13 | 98 | 8 | 15 |
| Opunohu | 2 | 7 | 17 | 2 | 2 | 0 | 4 | 20 | 85 | 7 | 70 |
| Opunohu | 2 | 8 | 22 | 3 | 3 | 1 | 7 | 20 | 150 | 9 | 32 |
| Opunohu | 2 | 9 | 18 | 4 | 1 | 1 | 6 | 24 | 211 | 11 | 31 |
| Opunohu | 2 | 10 | 26 | 3 | 1 | 1 | 5 | 13 | 90 | 8 | 15 |
| Opunohu | 2 | 11 | 18 | 2 | 1 | 0 | 3 | 24 | 802 | 11 | 102 |
| Opunohu | 2 | 12 | 37 | 4 | 3 | 1 | 8 | 10 | 69 | 9 | 16 |
| Opunohu | 2 | 13 | 34 | 9 | 4 | 1 | 14 | 14 | 65 | 5 | 22 |
| Opunohu | 2 | 14 | 20 | 5 | 1 | 0 | 6 | 20 | 270 | 12 | 153 |
| Opunohu | 2 | 15 | 21 | 2 | 4 | 0 | 6 | 11 | 60 | 14 | 43 |
| Opunohu | 2 | 16 | 19 | 4 | 1 | 0 | 5 | 14 | 160 | 10 | 55 |
| Opunohu | 2 | 17 | 25 | 2 | 2 | 0 | 4 | 20 | 185 | 7 | 34 |
| Opunohu | 2 | 18 | 27 | 2 | 0 | 0 | 2 | 105 | 629 | 8 | 15 |
| Opunohu | 2 | 19 | 32 | 4 | 5 | 1 | 10 | 16 | 87 | 9 | 50 |
| Opunohu | 2 | 20 | 35 | 3 | 3 | 0 | 6 | 20 | 109 | 11 | 98 |
| Opunohu | 2 | 21 | 19 | 1 | 0 | 0 | 1 | 13 | 95 | 23 | 23 |
| Opunohu | 2 | 22 | 25 | 0 | 2 | 0 | 2 | 20 | 108 | 27 | 34 |
| Opunohu | 2 | 23 | 32 | 3 | 3 | 0 | 6 | 15 | 311 | 9 | 246 |
| Opunohu | 2 | 24 | 17 | 5 | 2 | 0 | 7 | 16 | 237 | 9 | 57 |
| Opunohu | 3 | 1 | 13 | 0 | 1 | 0 | 1 | 15 | 107 | 26 | 26 |
| Opunohu | 3 | 2 | 12 | 1 | 1 | 0 | 2 | 14 | 143 | 13 | 19 |
| Opunohu | 3 | 3 | 15 | 1 | 2 | 0 | 3 | 18 | 94 | 16 | 23 |
| Opunohu | 3 | 4 | 23 | 5 | 2 | 0 | 7 | 19 | 171 | 14 | 85 |
| Opunohu | 3 | 5 | 16 | 1 | 2 | 1 | 4 | 11 | 52 | 1 | 7 |
| Opunohu | 3 | 6 | 36 | 3 | 1 | 1 | 5 | 16 | 86 | 10 | 18 |
| Opunohu | 3 | 7 | 17 | 2 | 2 | 1 | 5 | 10 | 66 | 10 | 59 |
| Opunohu | 3 | 8 | 22 | 5 | 3 | 1 | 9 | 17 | 125 | 8 | 15 |
| Opunohu | 3 | 9 | 18 | 4 | 3 | 2 | 9 | 51 | 128 | 17 | 70 |
| Opunohu | 3 | 10 | 26 | 2 | 2 | 1 | 5 | 14 | 75 | 3 | 16 |
| Opunohu | 3 | 11 | 18 | 2 | 1 | 1 | 4 | 13 | 368 | 11 | 25 |
| Opunohu | 3 | 12 | 37 | 5 | 4 | 2 | 11 | 9 | 28 | 6 | 10 |
| Opunohu | 3 | 13 | 34 | 9 | 5 | 2 | 16 | 9 | 51 | 2 | 19 |
| Opunohu | 3 | 14 | 20 | 5 | 1 | 0 | 6 | 13 | 65 | 10 | 49 |
| Opunohu | 3 | 15 | 21 | 3 | 1 | 0 | 4 | 11 | 73 | 11 | 29 |
| Opunohu | 3 | 16 | 19 | 3 | 1 | 0 | 4 | 14 | 109 | 6 | 10 |
| Opunohu | 3 | 17 | 25 | 3 | 2 | 1 | 6 | 12 | 123 | 9 | 78 |
| Opunohu | 3 | 18 | 27 | 2 | 0 | 0 | 2 | 48 | 209 | 11 | 15 |
| Opunohu | 3 | 19 | 32 | 6 | 4 | 1 | 11 | 11 | 40 | 9 | 17 |
| Opunohu | 3 | 20 | 35 | 3 | 3 | 0 | 6 | 15 | 143 | 11 | 47 |
| Opunohu | 3 | 21 | 19 | 1 | 0 | 0 | 1 | 16 | 105 | 10 | 10 |
| Opunohu | 3 | 22 | 25 | 1 | 1 | 0 | 2 | 15 | 270 | 14 | 21 |
| Opunohu | 3 | 23 | 32 | 1 | 1 | 0 | 2 | 13 | 109 | 17 | 45 |
| Opunohu | 3 | 24 | 17 | 4 | 1 | 0 | 5 | 16 | 68 | 8 | 57 |
